# Investigation of Nucleation Mechanism and Tapering Observed in ZnO Nanowire Growth by Carbothermal Reduction Technique

**DOI:** 10.1007/s11671-010-9738-3

**Published:** 2010-08-19

**Authors:** Ayan Kar, Ke-Bin Low, Michael Oye, Michael A Stroscio, Mitra Dutta, Alan Nicholls, M Meyyappan

**Affiliations:** 1 Electrical and Computer Engineering Department, University of Illinois, Chicago, IL 60607, USA; 2 Department of Physics, University of Illinois, Chicago, IL 60607, USA; 3 Department of Bioengineering, University of Illinois, Chicago, IL 60607, USA; 4 Research Resources Center, University of Illinois, Chicago, IL 60607, USA; 5 Center for Nanotechnology, NASA Ames Research Center, Moffett Field, CA 94035, USA

**Keywords:** ZnO, Nanowires, Microscopy

## Abstract

ZnO nanowire nucleation mechanism and initial stages of nanowire growth using the carbothermal reduction technique are studied confirming the involvement of the catalyst at the tip in the growth process. Role of the Au catalyst is further confirmed when the tapering observed in the nanowires can be explained by the change in the shape of the catalyst causing a variation of the contact area at the liquid–solid interface of the nanowires. The rate of decrease in nanowire diameter with length on the average is found to be 0.36 nm/s and this rate is larger near the base. Variation in the ZnO nanowire diameter with length is further explained on the basis of the rate at which Zn atoms are supplied as well as the droplet stability at the high flow rates and temperature. Further, saw-tooth faceting is noticed in tapered nanowires, and the formation is analyzed crystallographically.

## Introduction

Interest in nanowires continues to grow fueled by applications in electronics, optoelectronics, sensors, piezoelectric and thermoelectric devices, and energy storage [1]. In spite of considerable advances in growth and application development of nanowires, the various proposed growth mechanisms are still controversial and subject to immense discussion. For example, it is well documented that the diameter of nanowires grown via the vapor–liquid–solid (VLS) mechanism is determined by the size of the droplet. This is true but it does not necessarily imply that the diameter of nanowires is constant along its axis. It has been recently reported that the dynamic reshaping of the catalyst particles during the nanowire growth determines the length and shape of the nanowires [2]. Also, just as in elemental semiconductors [3], there is a general consensus that even for oxide nanowires the whole molten alloy particle, referred to as the catalyst, rises above the surface of the substrate and rides at the tip of the nanowire during the growth process. The objective of this paper is twofold. First, we investigate the initial stages of nucleation, oxidation of Zn atoms, and growth of ZnO nanowires. Secondly, we investigate the formation of tapered nanowires from the growth kinetics point of view. Compared to elemental and III–V nanowires, growth behavior of semiconducting oxide nanowires, and in particular ZnO is not well understood [1]. ZnO has been proven to be quite a complex and interesting material with a variety of structures such as nanowires, nanobelts, and tetrapods [4]. Each of these structures can be formed by different growth mechanisms under widely different thermodynamic conditions. The recent surge in applications of ZnO nanowires as a piezoelectric material [5] for energy harvesting has led to the present investigation. The dependence of nanowire diameter on the amount of generated piezoelectricity requires clarification of the role of gold at the nanowire tip in controlling the shape and diameter of the nanowire [6].

## Experimental Work

The source consists of zinc oxide (ZnO) metal basis of 99.999% purity mixed with graphite in a weight ratio of 1:1 to carry out a carbothermal reduction process. A 1" diameter quartz tube was inserted inside an isothermal furnace, and the source mixture was kept in a quartz boat inside this tubular reactor. Gold colloids were used as the catalyst for different experiments. The substrate with the Au catalyst was placed downstream from the quartz boat located at the center of the heating zone. One end of the quartz tube was connected to a mass flow controller, which controls the flow rate of the carrier gas, argon, and the other end was connected to the exhaust. Nanowires were grown within a temperature window of 900–980°C, whereas the carrier gas flow rate was varied between 100–160 sccm.

Fifty nanometres Au colloids (BBI International) were used as the starting catalyst. The c-sapphire substrate was treated with poly-L-Lysine before dropcasting the Au colloidal solution. The substrate was then spun at 2000 rpm for 1.5–2 min ensuring a random dispersion of the gold particles. The flow rate of Ar carrying the Zn vapor was maintained at 100 sccm and turned on only for the duration of growth after the temperature was allowed to stabilize at 900°C. At the synthesis temperature, carbothermal reduction of the ZnO powder yields Zn vapor according to the following reactions:

(1) Carbothermal Reduction (source):ZnO(s)+C(s)→Zn(v)+CO(g)

(2) Catalystalloy formation:Zn(v)+Au(s)→Au−Zn(l)

## Results and Discussion

**Figure 1:**
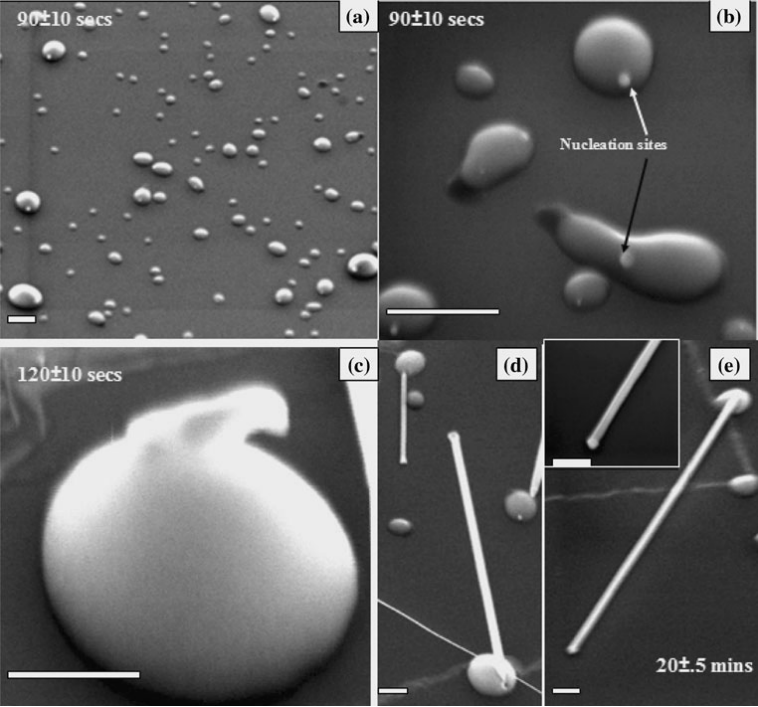
**a and b show the Au–Zn clusters with the nucleation sites formed on the substrates**. **c** Early stage of nanowire nucleation from the Au–Zn clusters. **d** and **e** show eventual wire formation with the presence of the catalyst at the nanowire tip (also shown as inset in fig **e**). *Scales* in figure (**a**), (**d**), and (**e**) are 2 μm, whereas in figures (**b**) and (**c**), the scale is 500 nm and 200 nm, respectively.

**Figure 2:**
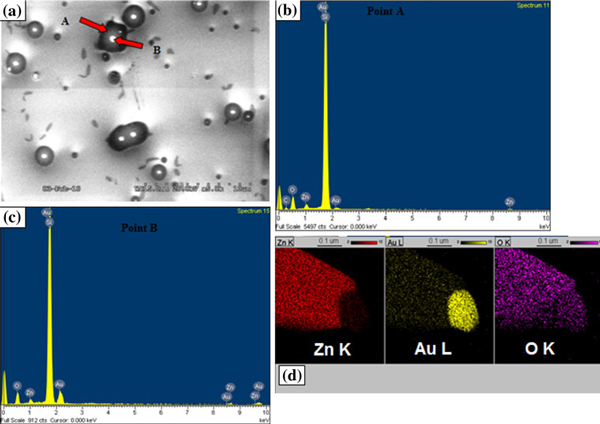
**a SEM image of the Au–Zn nucleation sites formed on the substrates at early stages of growth**. **b** and **c** SEM-EDX done on point *A* and point *B* as marked in figure (**a**). **d** shows STEM elemental spectral analysis done on an individual nanowire.

Figure 1a shows the SEM image of the substrate surface after the gold nanoparticles were exposed to the ZnO:C source at 900°C for *t* = 90 ± 10 s. In the initial stages of temperature ramping, the substrate becomes covered with Au islands, which become the preferential sites for Zn incorporation. The Zn atoms can either condense from the vapor phase or be transferred from adjacent regions of the substrate. Then, they rapidly diffuse into the Au clusters forming the Au–Zn clusters. The Zn concentration in these particles increases with time, until a solid crystal nucleates out of the alloy droplet due to supersaturation in the droplet. SEM-EDX was performed in order to investigate the islands seen in Figure 1a, b and to gain further insight about the nucleus formation. Figure 2a shows a SEM image of the substrate surface after *t* = 90 ± 10 s. The EDS measurement was performed on points A and B of the island in Figure 2a, and the acquisition time was 120 s. The major signals from EDS are O (Kα) at 0.524 keV, Au (Mα) at 2.1 keV, and Zn (Lα) at 3.4 keV using a 20 kV electron beam. The data obtained shows that the island (point A) has a greater concentration of Zn compared to Au. With increasing amount of Zn condensation and dissolution from the source vapor, Zn and Au form an alloy and liquefy giving rise to the islands on the substrate. This leads to an increase in the volume of the alloy droplet as more Zn is carried to the droplet and dissolves. The process of Zn dissolution into the alloy continues till the ratio of Zn in the Au–Zn alloy increases beyond a certain thermodynamic limit, leading to the formation of the nucleus. In contrast to the islands, the nucleus has more Au compared to Zn as in the EDX for point B. This is further confirmed by the STEM-XEDS elemental mapping of a single ZnO wire (see Figure 2d), which shows a high Au concentration (yellow) in the catalyst at the tip of the nanowire. As Zn continues to further condense/dissolve into the nucleus, precipitation of the ZnO nanowire would start underneath this nucleus with Au-dominated nucleus riding on top of the nanowire as catalyst. Hence, the Au/Zn ratio is found to vary from the islands remaining on the substrate to the nucleus that becomes the catalyst riding on top of the wire.

Formation of a crystal nucleus from the liquid Zn–Au metal droplet (schematic of which is shown later in Figure 4a) leading to nanowire growth is shown in two stages in Figure 1b, c for *t* = 90 ± 10 and 120 ± 10 s, respectively. Thermodynamically, Gibbs free energy minimization is the criterion to be satisfied for the formation of the nucleus. After the nucleus is formed, ZnO nanowires start to form, as shown in Figure 1d, e for *t* = 18 ± 1 min and 20 ± 0.5 min, respectively, with the catalyst at the tip which acts as the sink for the Zn atoms and generates a concentration gradient along the catalyst particle. However, the Zn–Au alloy droplet on which the nuclei forms remains on the substrate. The nanowire formation can be described by the following reaction

(3) $$\text{Nanowire growth: Au}-\text{Zn}(l)+\text{Zn}(v)+1/2{\text{O}}_{2}\to \text{Au}-\text{Zn}(l)+\text{ZnO}(s)$$

The oxidation process of the Zn leading to the formation of ZnO contributing to the nanowire growth is a critical step. In the present case, it is likely that ZnO nanowires originate from the oxidation of the Zn atoms within the Au–Zn alloy particle since it is commonly known that the activity of metals can be increased upon alloying. It has been previously reported that the oxidation of alloys such as Zn–Ag and Zn–Cu results in the formation of ZnO crystalline precipitates [7]. In a similar manner, it is believed ZnO will precipitate in the form of ZnO nanowires due to the oxidation of Au–Zn particles. There have also been recent claims stating that there is no involvement of a liquid or solid catalyst at the tip of ZnO nanowires in the growth process involving thermal evaporation [8]. The SEM images in Figures 1d, e and 2d here show a distinct catalyst at the nanowire tip that serves to control the diameter of the nanowire.

We propose here that Zn atoms are the main species contributing to crystal growth and not ZnO. The gold particles alloy with Zn and the oxide nanowires grow with the assistance of the liquid catalyst particles at the wire tip where the Zn atoms are oxidized, as discussed previously. Since the ZnO crystal nucleates at the solid–liquid interface, the nanowire diameter is determined and controlled by the size of the gold catalyst droplet at the tip, which is a feature common in VLS process [1,9]. We also point out contradicting reports [10] wherein nanowire branches with substantially different diameters compared to the catalyst particle have been seen; a growth mechanism different from VLS was suggested, and ZnO atoms were believed to be the source of nanowire growth instead of Zn atoms.

**Figure 3:**
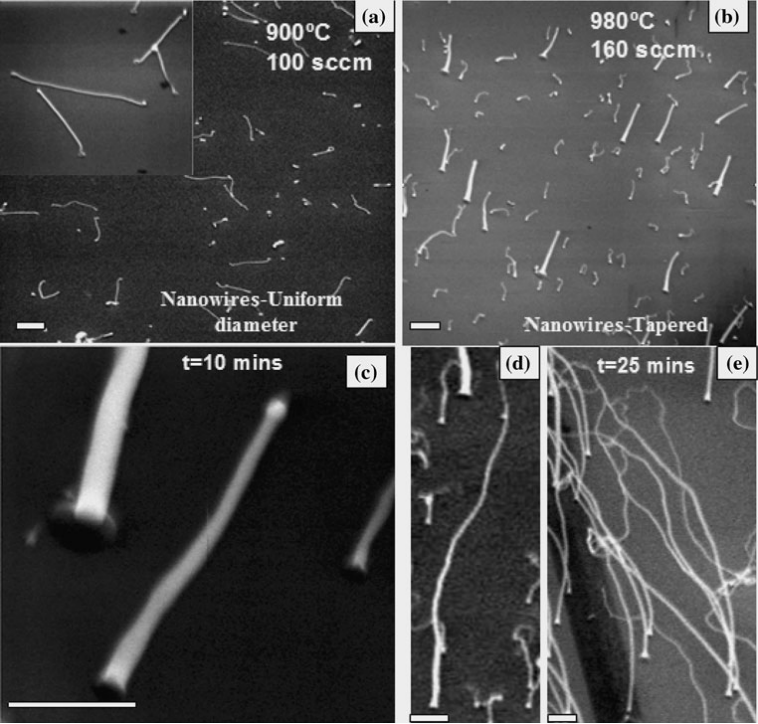
**a ZnO nanowires with uniform diameters grown on p-type Si (100) substrates**. **b**–**d** show nanowires with a constant decrease in diameter along the axis grown at a higher flow rate and temperature. The growth time for figures (**b**) and (**c**) were 10 min and that for figures (**e**) and (**f**) were 25 min. Scales in all the figures are 2 μm.

**Figure 4:**
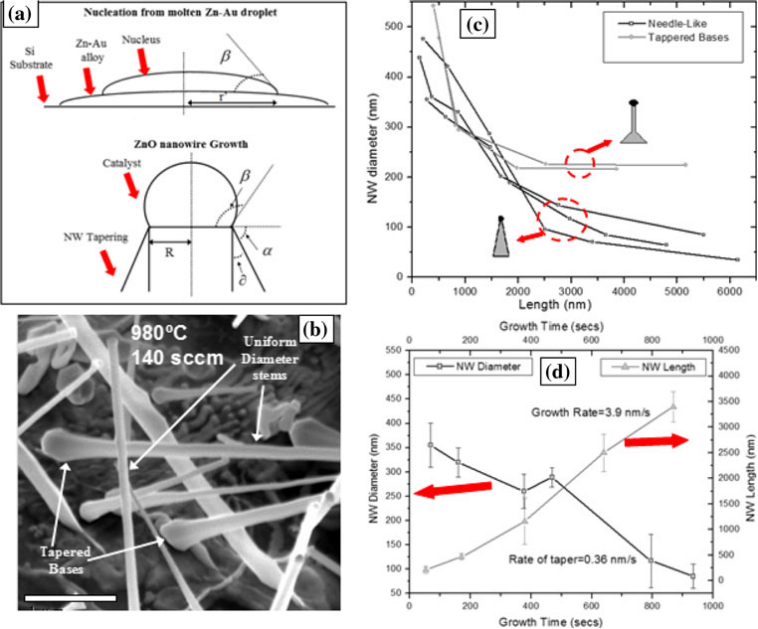
**a Schematic of the nucleus formed on the molten Au–Zn droplet and ZnO nanowire growth at initial stages with tapering**. **b** ZnO nanowires with tapered base and uniform long stems. **c** Plot of nanowire diameter vs. length for both tapered and needle-like ZnO nanowires. **d** Plot showing growth and tapering rates of needle-like ZnO nanowires.

The shape of the semiconductors at the nanoscale is another decisive factor for the properties, and the shape controlled growth of semiconductors can find unique applications in electronics and photonics. Until now, tapered ZnO nanowires have only been produced by chemical synthesis or electrochemical deposition method [11]. Here, we report the observation of tapered ZnO nanowires grown using the carbothermal reduction method in a furnace. Figure 3a shows nanowires with a uniform diameter throughout their axis, grown on p-type silicon substrates at a temperature of 900°C and argon flow rate of 100 sccm. Tapered nanowires are formed at a growth temperature of 980°C and an Ar flow rate of 160 sccm as seen in Figure 3b–e. The growth times were 10 min for Figure 3b, c and 25 min for Figure 3d, e. At a lower flow rate of 140 sccm but at the same temperature of 980°C, nanowires with just a tapered base but uniform long stems are seen in Figure 4b. The illustration in Figure 4a is used in order to understand the tapering mechanism. To put things into perspective, the growth of tapered nanowires can be categorized as a special case of cylindrical nanowires with the flank angle *δ* = 0. In fact, diameters of epitaxially grown Si nanowires have been shown [12] to vary, especially in the region close to the substrate where the nanowires exhibit larger diameters just as seen here for ZnO nanowires in Figures 3b–e and 4b. One first guess would be that this large diameter is created by radial overgrowth of the nanowire after axial growth. Especially at elevated temperatures, surface diffusion and vapor-solid growth might influence the shape and result in enlargement of the nanowire base. In particular, a faceting of the nanowire base expansion, often observed at high temperatures, might occur after growth by surface diffusion. Such faceting of the base is seen in Figure 4b as indicated by arrows.

The illustration in Figure 4a shows that the decrease in nanowire diameter along its length is controlled by the size of the catalyst contact area at the liquid–solid interface. In our case, the angle *α* equals to zero at the beginning stages of nucleation. As growth begins, the angle *α* has to increase, which is accompanied by an increase in the contact angle *β* based on a modified Young's equation [12]:

(4) $$\sigma_{l}\cos(\beta )=\sigma_{s}\cos(\alpha )-\sigma_{ls}-\frac{\tau }{R}$$

which means that the droplet approaches a larger solid angle of spherical section. Here, *σ*_*l*_, *σ*_*ls*_, and *σ*_*s*_ are the surface tension of the droplet surface, the liquid–solid interface, and the solid, respectively. The droplet at the tip of the nanowire has a contact area of radius *R* and *τ* is the line tension. The increase in the contact angle *β* causes a decrease in the contact area and a decrease in the radius R of the droplet. Consequently, the radius of the nanowire *R* should be smaller than the initial radius *r*' of the contact area of the nuclei on the Au–Zn droplet. Thus, the nanowire diameter is largest at the base and decreases as the length of the wire increases, with the diameter being directly controlled by the shrinking radius of the contact area *R* at the droplet tip. The average rate of decrease in the nanowire diameter with time (tapering rate) for needle-like nanowires here is estimated as 0.36 nm/s and plotted in Figure 4d. Also, plotted is the growth rate of the nanowires, and the average rate throughout the process is estimated as 3.9 nm/s. However, the growth rate is relatively slower at the onset of growth and speeds up with time. The decrease in diameter of the nanowire with length of both needle-like nanowires and the ones with just tapered bases are plotted in Figure 4c. The rate of decrease in diameter is much larger close to the base and then decreases as the length increases. This can be explained by the fact that the rate of increase of angle *α* with length is large close to the base of the nanowire and decreases gradually. Thus, one reason for the decrease in nanowire diameter with length, i.e. tapering, is the reduction in the size of the catalyst at the tip as growth progresses.

Nanowires of two different morphologies are recalled here: in one case, we have the tapering just restricted to the base, as seen in Figure 4b, and another where we see a continuous decrease in nanowire diameter with length, as in Figure 3b–e; both morphologies can be explained on the basis of the rate at which the Zn atoms are deposited/delivered to the substrate if the temperature is kept constant. The concentration of the Zn adatoms at the substrate will increase as the Ar carrier gas flow rates are increased. Due to the concentration gradient between the substrate surface and the nanowire, diffusion of the adatoms becomes prominent at flow rates >140 sccm. Excess growth species are available at the base of the ZnO nanowire, where the mobility of Zn atoms diffusing from the substrate surface to the nanowire tip is impeded and allows radial tapering of the base [13]. The base diameter thus increases steadily with an increase in flow rates. This gives rise to tapered, as in Figure 4b, but not needle-shaped nanowires, as in Figure 3. Further, it is to be noticed that the tapered segment of the nanowire in Figure 4b is very small. This is probably due to the limitation of adatom diffusion via the nanowire sidewall, since the upward adatom mobility via the nanowire sidewalls decreases at high temperatures [14]. However, as the flow rates are increased further, the tapered nanowires give way to needle-like nanowires, obtained in this case at a flow rate of 160 sccm as shown in Figure 3b–e where there is a constant decrease in diameter with length. Such observations have been reported previously in the growth of InAs nanowires [13]. The tapering also indicates that adatom surface diffusion from the substrate up the nanowire sidewalls forms a path for the growth species reaching the alloy droplet other than the direct impingement of the atoms on the droplet, as has been reported previously [13]. A theoretical study [15] investigating the shape of the nanowires on the basis of the contact angle *β* of the liquid droplet at the nanowire tip found surprisingly that tapered nanowire growth (∂ > 0) is more likely for a wide range of contact angles *β* compared to nanowires with uniform diameters.

**Figure 5:**
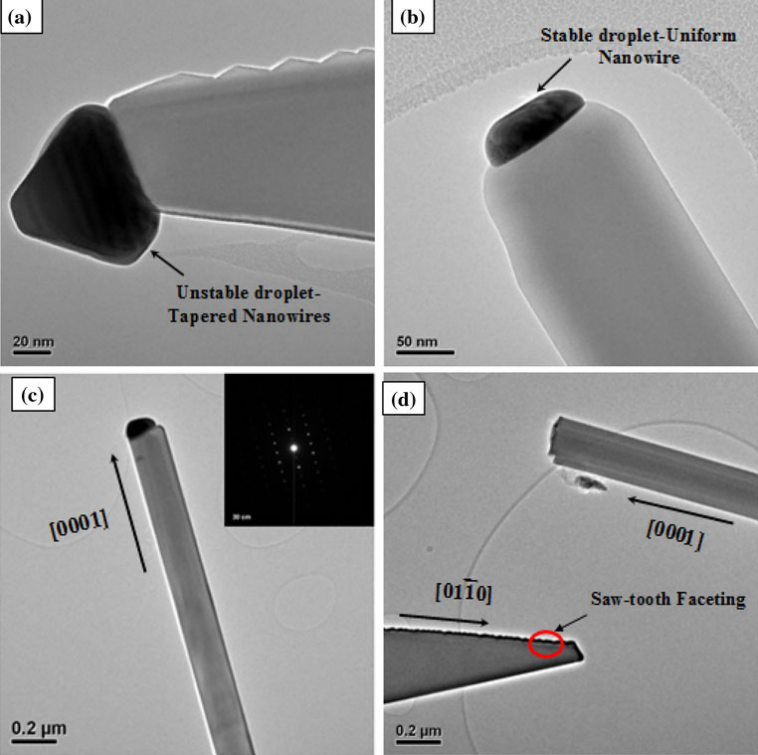
**TEM images of (a) tapered ZnO nanowires (b, c) nanowire with uniform diameter, inset in figure c shows diffraction pattern**. **(d)** TEM image of straight and tapered nanowires indicating the growth directions.

The tapering of the nanowires can also be explained on the basis of the stability and shape of the Au catalyst at the tip of the nanowires. Nanowires with uniform diameters, seen in Figures 1 and 3a, are formed when the growth species land at a constant rate on the droplet, and the droplet is sufficiently stable (when surface tension > stress) at the growth temperature to resist decay or disintegration and hence maintaining its spherical shape. However, it has been noted previously [16] that high temperatures and flow rates cause droplet instability, which may be one of the reasons for the continuous decrease in nanowire diameter with length. Mohammad reported [16] that high temperatures and flow rates cause the Au particle at the tip of the nanowire to lose its spherical shape indicating an unstable droplet. This droplet instability leading to the formation of tapered nanowires is clearly seen in Figure 5, which compares the shape of the catalyst at the tips of tapered (Figure 5a) and straight (Figure 5b) nanowires. The catalyst at the tip of the tapered nanowire has an ellipsoidal/triangular kind of shape, whereas the straight nanowire still maintains a hemispherical particle shape. It appears that the catalyst in the tapered wire is elongated along the axial direction, and it is not clear whether this elongation is an intermediate state of the particle during the course of the shrinking or if it represents some quasi-equilibrium shape at the experimental temperature and flow rate. Figure 5c shows a representative straight wire with its corresponding diffraction pattern, which also reveals a zone axis of $\left[\bar{2}110\right]$. The catalyst particle can also be seen at the end of the wire, which is spherical in shape. For the straight wire, the growth is in the [0001] direction, with both edges of the wires possibly bounded by the $\left(01\bar{1}0\right)$ plane. Figure 5d shows TEM images of the straight and tapered wires where the straight wire is found to be growing along the *c*-axis, whereas the tapered wire along the $\left[01\bar{1}0\right]$ direction.

Oversupply of source vapor as well as interplay of surface energies of the wire and liquid droplet was also reported to cause the droplet to be unstable, leading to oscillations in resulting Si NW structures [17]. This is seen here in the case of the tapered ZnO wire in Figure 5d, and such oscillations of the droplet resulting in faceting in the nanowire sidewalls is discussed below in detail. To our knowledge, this is the first observation of periodic saw-tooth faceting in ZnO nanowires; the observed faceting is periodic, where the period P of the facets is about 19.2 nm on average and the height *H* is about 4 nm. Incidentally, such faceting is observed only in tapered nanowires and not in straight ones as seen in Figure 5d, indicating such faceting might have some relation to the tapering mechanism observed in our case.

**Figure 6:**
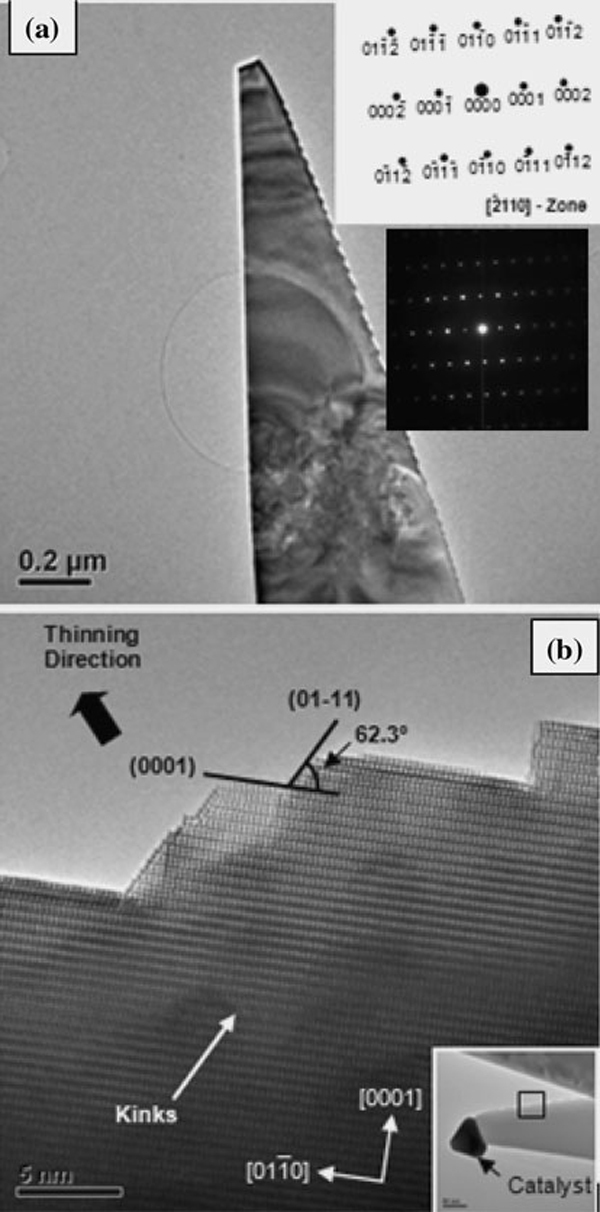
**a and b shows TEM and HRTEM images of saw-tooth faceting observed in tapered nanowires, respectively**.

Figure 6a shows a tapered wire and its electron diffraction pattern, which indicates that it is mono-crystalline. The zone axis of the diffraction pattern is the $\left[\bar{2}110\right]$ of the hexagonal ZnO. The base of these tapered wires varies greatly from half a micron to a few microns in size. However, it is important to stress that TEM observations may not provide an accurate estimation of the wire dimensions due to the nature of sample preparation. Although the lateral sizes of these wires are in the sub-micrometer scale, they still possess moderate degree of electron transparency at low magnification, indicating that these wires have somewhat plate-like morphology. In other words, the widths of these wires are in the sub-micron length scale; if the wires are hypothesized to have thicknesses similar to their widths, then there will be no electron transparency even in a 300 kV TEM. Given that atomic resolution was observed on these wires, the wire thicknesses must be in the range of few tens of nanometers, which means they are thin and wide (plate-like).

**Figure 7:**
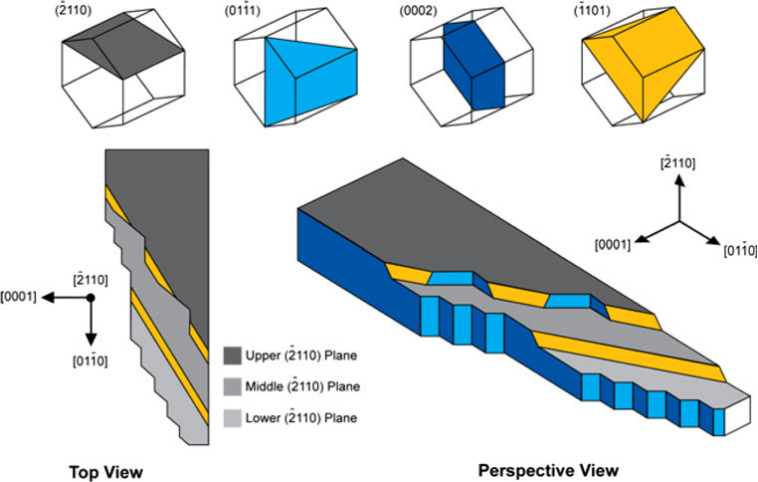
**Model correlating the growth of tapered nanowire with saw-tooth faceting based on crystallography**.

The tapered wire in Figure 6a reveals saw-toothed morphology only on one of the edges, while the other edge is atomically smooth. According to its diffraction pattern, the smooth edge of the wire is running along the $\left[01\bar{1}0\right]$ direction, and it is hypothesized that this edge is exposing the {0002} plane. High-resolution images taken from another tapered wire Figure 6b confirms that the saw-toothed morphology is representative. Figure 6b inset shows the location on the tapered wire from which the high-resolution images were obtained. Note that the catalyst particle (darker contrast) is still intact on the tapered wire. Figure 6b shows that the saw-toothed edge consists of two predominant types of planes: the terraces, which are parallel to the smooth edge, are exposing the (0002) plane; the steps, which consistently make an angle of 62.3° (theoretical: 61.4°) with the (0002), are exposing the $\left(01\bar{1}0\right)$ plane. It is important to note that strong lattice fringes are only visible at the tapered, saw-toothed edge, suggesting that the wire's cross-section is not uniform but actually wedge-like, thinning toward the tapered, saw-toothed edge. Based on the observed preferential exposure of the {0002} and $\left\{01\bar{1}1\right\}$-type planes in the hexagonal ZnO crystal, a model in Figure 7 was constructed to describe the growth of the tapered wire in relation with its crystallographic orientation. We have seen that steps exposing the $\left(01\bar{1}1\right)$ plane help mitigate the tapering along the edge of the wire. Given that there are other crystallographically equivalent $\left\{01\bar{1}1\right\}$ planes in the hexagonal system, the model proposes that atomic-scale steps should also exist on the top and bottom surfaces of the wire. The possible step configurations are illustrated in the diagram: (1) pure $\left(\bar{1}101\right)$ steps and (2) $\left(\bar{1}101\right)$ steps with (0002)/$\left(01\bar{1}1\right)$ kinks. Indeed, the orientations of the 'kinks', indicated in Figure 6d, agree qualitatively with the proposed step configurations on the surfaces of these wires.

One mechanism that could lead to such faceting is surface reconstruction due to charge stabilization in polar compounds. ZnO crystal is formed by alternating stacks of oppositely charged O^2-^ and Zn^2+^ planes parallel to the surface. If the resulting dipole moment perpendicular to the surface is nonzero, stabilization of such a surface is accomplished by a rearrangement of surface charges or by introducing compensating charges into the outermost surface planes [18]. This could lead a significant modification of the surface geometric structure and stoichiometry. The stabilization mechanism for the Zn-terminated face of ZnO has been experimentally investigated by a variety of techniques, and various mechanisms have been proposed for the reduction in the surface charge density to yield a stable Zn termination. Recent experimental studies [19] combined with theoretical calculations [20] suggest that the Zn-terminated surface can be stabilized by a reconstruction involving triangular surface structures [21].However, due to the unstable nature of the catalyst at the wire tip, it is believed here that faceting due to droplet oscillation is the dominant mechanism that causes the surface reconstructions. The observations seen here can be explained based on a thermodynamic model used earlier to explain similar faceting in Si nanowires [17]. The allowed facets correspond to a wire that is widening or narrowing as it grows. The wire widens as the droplet is stretched thinner and contact angle *β* (Figure 4a) decreases, which generates an inward force favoring the introduction of the narrowing facet. Conversely, the narrowing of the wire leads to the droplet applying an increasing outward force on the wire, favoring an introduction of the widening facet. This oscillatory mechanism leads to the periodic faceting seen here.

## Concluding Remarks

In this investigation, using the carbothermal reduction technique, ZnO nanowires are found to grow from nuclei on the molten Au–Zn clusters. A tapering of diameter along the axis is observed with the largest diameter at the base. This may be due to the change in the contact angle *β* of the catalyst droplet at the nanowire tip causing a change in the contact area R at liquid–solid interface. The growth rate of the needle-like nanowires is found to be 3.9 nm/s and the tapering rate is established to be 0.36 nm/s on the average. Finally, the rate of addition of Zn atoms is found to control the tapering where the tapering limited to the nanowire base gives way to a continuous taper along the nanowire length at higher deposition rates. The tapered wires are also found to have unstable droplets at their tips, which are believed to be a cause of tapering.
